# Wie ältere Menschen durch KI-gestützte Gesundheitstechnologien lernen: Von süßen Robben und nervösen Sturzsensoren

**DOI:** 10.1007/s00391-025-02425-5

**Published:** 2025-03-27

**Authors:** Katrin Lehner, Vera Gallistl, Rebekka Steinlechner, Sophie Kellerberger, Gerhard Paulinger, Franz Kolland

**Affiliations:** 1 Kompetenzzentrum Gerontologie und Gesundheitsforschung, Karl Landsteiner Privatuniversität für Gesundheitswissenschaften, Krems an der Donau, Österreich; 2 Dr.-Karl-Dorrek-Straße 30, 3500 Krems, Österreich

**Keywords:** Altenpflege, Geragogik, KI-Kompetenz, KI-Sturzsensoren, Roboter, Geriatric nursing, Geragogy, AI literacy, AI fall detection sensors, Robots

## Abstract

**Hintergrund:**

Mit dem wachsenden Einsatz von künstlicher Intelligenz (KI) in verschiedenen Lebensbereichen werden vermehrt auch KI-Technologien für die Pflege und Betreuung älterer Menschen entwickelt. Forschungen zu Einstellungen und Praktiken pflegebedürftiger älterer Menschen zu KI stehen derzeit noch am Beginn.

**Ziel der Arbeit:**

Ziel des Beitrags ist es, danach zu fragen, welche Lernprozesse bei älteren Pflegeheimbewohner*innen durch den Einsatz von KI-Technologien angestoßen werden, und wie diese geragogisch unterstützt werden können.

**Material und Methoden:**

Die Datenbasis stellen 10 leitfadengestützte Interviews mit älteren Heimbewohner*innen (83 bis 96 Jahre alt) sowie teilnehmende Beobachtungen (75 h) dar. Die Erhebung fand im Sommer 2022 und Herbst 2023 in 2 Pflegeheimen statt; diese setzen KI-basierte Sturzsensoren und soziale Roboter ein. Das Material wurde mithilfe der Situationsanalyse ausgewertet.

**Ergebnisse:**

Ergebnisse zeigen, dass Pflegeheimbewohner*innen ein aktives Interesse an der Auseinandersetzung mit KI-Technologien haben – etwa, indem sie versuchen, Erfahrungen mit KI-Technologien mit Erfahrungen ihres Lebenslaufs zu verbinden (*biografieorientierte Lernpraktiken*) und sich im Alltag mit KI-Technologien auseinandersetzen (*alltagsorientierte Lernpraktiken*). Bewohner*innen sind also interessierte Akteur*innen in Prozessen der Technikimplementierung, nehmen sich allerdings selbst nur selten als kompetent im Kontext von KI wahr.

**Diskussion:**

Der Beitrag zeigt die Lernpraktiken und -potenziale der Bewohner*innen und diskutiert Möglichkeiten eines geragogischen Zugangs für ein differenziertes Verständnis von KI-Technologien in der Pflege.

## Abstract (en)

**Background:**

With the growing use of artificial intelligence (AI) in various areas of life, AI technologies are increasingly being developed for the nursing and care of older people and are intended to contribute to greater safety for older people in need of care and to relieve the burden on caregivers; however, research into the attitudes and practices of older people in need of care towards AI is only just beginning.

**Objective:**

The aim of the article is to ask which learning processes emerge as older nursing home residents interact with AI technologies and how these can be supported with concepts from geragogy.

**Material and methods:**

The data consist of 10 guideline-assisted interviews with older nursing home residents aged 83–96 years and records of participant observations (75 h). Data collection took place in summer 2022 and fall 2023 in two nursing homes that use AI-based fall sensors and social robots. The material was analyzed using situation analysis.

**Results:**

Nursing home residents showed an active interest in engaging with AI technologies, for example, by trying to relate experiences with AI technologies with experiences from their life course (biography-oriented learning practices) and engaging with AI technologies in everyday life (everyday-oriented learning practices). Residents are therefore interested actors in processes of technology implementation but rarely perceive themselves as competent in the context of AI.

**Conclusion:**

The article shows the learning practices and potentials of residents and discusses possibilities of a geragogical approach for a differentiated understanding of AI technologies in nursing.

Technologien in der Pflege von älteren Menschen basieren zunehmend auf künstlicher Intelligenz (KI). Der Fokus des Entwicklungsprozesses liegt häufig auf der lernenden Maschine statt auf den Lernprozessen älterer Nutzer*innen. Dieser Beitrag fragt danach, welche Lernprozesse bei älteren Pflegeheimbewohner*innen stattfinden, wenn KI-Technologien in der Pflege eingesetzt werden. Ausgehend von den Ergebnissen werden Anschlusspunkte für eine geragogische Unterstützung der KI-Entwicklung und -Implementierung in der Pflege diskutiert.

## Hintergrund

Technische Systeme, die künstliche Intelligenz (KI) beinhalten – und damit sind algorithmische Systeme gemeint, die auf Basis von großen Datenmengen automatisierte Entscheidungen treffen – wurden in den letzten Jahren zunehmend für unterschiedliche Kontexte des Alter(n)s entwickelt [2, 22] und gelten als „dritte Generation“ von Ambient-Assisted-Living(AAL)-Technologien [2]. Anwendungsfelder von KI-Technologien im Kontext von Gesundheit und Pflege reichen von intelligenten Toiletten, Betten und Sturzsensoren bis hin zu Smart Homes und assistiven Robotern.

Während Forschung und Entwicklung in diesem Bereich mit einer beachtlichen Geschwindigkeit voranschreiten, wurden die Perspektiven älterer Menschen auf das Thema KI in der Pflege nur selten untersucht [8]. Die Entwicklung von KI-Technologien ist in einen Alters- und Innovationsdiskurs eingebettet, der ältere Menschen als passive Nutzer*innen, denen aktive und lernwillige Technologien gegenübergestellt werden, positioniert [16]. Diese Engführung übersieht allerdings, dass sich ältere Menschen aktiv mit KI-Technologien auseinandersetzen und sich diese selbstbestimmt aneignen. Empirische Studien zum Einsatz von KI zeigen, dass es aufgrund von Stereotypen teilweise sogar vermieden wird, die Funktionsweise von KI-Technologien zu erklären und ältere Menschen so von der aktiven Nutzung ausgeschlossen werden [8]. Im Setting der Pflege kann das dazu führen, dass Lernprozesse vonseiten der Pflege- und Betreuungskräfte im Prozess der KI-Implementierung in den Blick genommen werden, nicht jedoch die der Bewohner*innen [18].

## Konzeptionelle Perspektive: Lernpraktiken im Kontext von künstlicher Intelligenz

Anknüpfend an geragogische Konzepte von alltagsnahem Lernen im Kontext von Technikentwicklung und -implementierung [21] wird in diesem Beitrag ein praxistheoretisches Konzept von Lernen im Alter verfolgt. Der analytische Fokus verschiebt sich von formalen oder nonformalen Lernumwelten hin zu lebensweltlichen Kontexten des Lernens im Alltag älterer Menschen [9]. Insbesondere im Kontext neuer Technologien sind solche alltäglichen und informellen Lernpraktiken bedeutend, da bestehende Forschung darauf hingewiesen hat, dass sich ältere Menschen Wissen zur Nutzung neuer Technologien häufig im Alltag, informell und manchmal sogar spielerisch („en passant“) aneignen [3, 24]. Solche und ähnliche Studien haben auch darauf verwiesen, dass der Widerstand gegenüber neuen Technologien als Teil eines selbstbestimmten, aktiven und lernenden Umgangs mit Digitalisierung verstanden werden kann [10].

Unter Lernpraktiken versteht der Beitrag nicht nur gezielte Formen der Aneignung von neuen Kompetenzen und neuem Wissen, sondern bezieht sich auf ein erweitertes Lernverständnis der Geragogik. Dieses schließt die Entwicklung von Reflexions- und Gestaltungskompetenz bewusst als Teil von Lernen ein [5] und versteht die Fähigkeit, sich selbstbestimmt an unterschiedliche, sich im Alternsprozess verändernde Lebensbedingungen anzupassen, als Kern von Lernprozessen im späteren Leben [12]. Erweitert wird ein solches geragogisches durch ein praxistheoretisches Verständnis von Lernen, das Lernpraktiken als Verkörperung sozialer Strukturen betrachtet [4], die mit Prozessen der Subjektivierung [1] einhergehen. Wir passen uns durch Lernprozesse nicht nur selbstbestimmt an sich verändernde Umweltbedingungen an, sondern verstehen durch Lernen auch, wer wir sind, und welche Positionen wir in dieser Welt einnehmen [2]. Dies ist v. a. bei neuen Technologien relevant, da die Auseinandersetzung mit und das Erlernen von digitalen Technologien von internalisierten Stereotypen und Selbstzuschreibungen älterer Menschen als uninteressiert und inkompetent beeinflusst wird [11]. Im Sinne der Geragogik lässt sich davon ausgehen, dass sich Lernen im Kontext von KI sowohl im Welt- als auch im Selbstbezug [6] vollzieht. Im Folgenden werden daher neben konkreten Lernpraktiken auch die mit diesen Lernpraktiken verbundenen Subjektivierungsprozesse herausgearbeitet, und es wird danach gefragt, inwiefern sich ältere Pflegeheimbewohner*innen als kompetente Akteur*innen verstehen, wenn sie mit KI-Technologien interagieren und diese erlernen.

Demnach stellen sich für die Analyse die folgenden Forschungsfragen:

- F1: Welche Lernpraktiken älterer Menschen zeigen sich im Umgang mit KI-Technologien im Kontext der Langzeitpflege?
- F2: Welche Formen der Subjektivierung (im Sinne von Selbstbeschreibungen im Kontext von KI) finden sich in Verbindung mit diesen Lernpraktiken?

## Studiendesign und Untersuchungsmethoden

Diese Arbeit untersucht den Einsatz von 2 KI-Systemen: einem KI-gestützten Sturzsensor und einer Roboterrobbe; diese stellen typische Anwendungsbereiche von KI in der Pflege dar [17]. Ersterer dient der Erkennung von Stürzen mithilfe von Tiefendaten, ist täglich in den Räumen der Bewohner*innen aktiv und soll Pflegepersonal im Fall eines Sturzes alarmieren. Die Roboterrobbe ist mit mehreren Sensoren ausgestattet und reagiert auf Berührung, Stimme und Licht in Form von Bewegungen, Geräuschen und Blinzeln. Bei regelmäßiger Verwendung lernt die Robbe durch KI, auf ihren Namen zu reagieren. In den untersuchten Einrichtungen findet der Roboter überwiegend in der Senior*innenbetreuung im Gruppen- und im Einzelsetting Einsatz.

Die Datenerhebung erfolgte mithilfe von 10 leitfadengestützten Interviews, welche zwischen 21 und 64 min andauerten und im Sommer 2022 und Herbst 2023 in 2 österreichischen Pflegeheimen durchgeführt wurden. Zusätzlich zu den Interviews wurden 75 h teilnehmende Beobachtung in den beiden Pflegeheimen durchgeführt und dokumentiert. Der Zugang zum Feld erfolgte durch die Heimleitungen, über die Auswahl der interviewten Personen entschied das Pflegepersonal, basierend auf dem Gesundheitszustand und der Tagesverfassung der Bewohner*innen. Die interviewten Bewohner*innen waren zwischen 83 und 96 Jahre alt und lebten zumindest ein Jahr in der jeweiligen Pflegeeinrichtung. Der verwendete Leitfaden thematisierte tägliche Routinen der Bewohner*innen, ihre Sicht auf den Einsatz der KI sowie mögliche Bias und Fehler der Technologien.

**Abb. 1:**
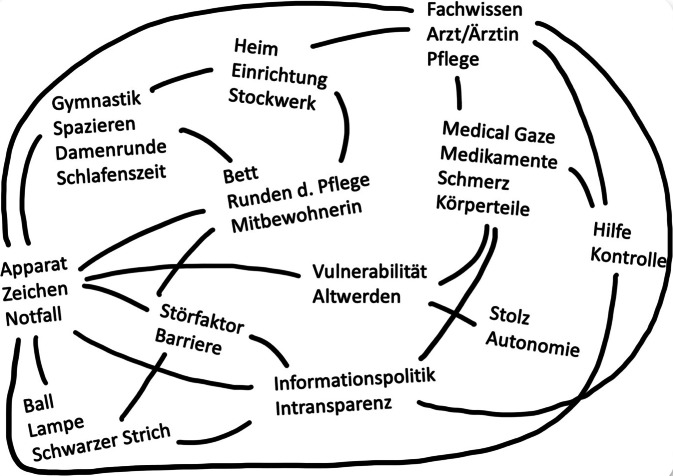
Exemplarische Darstellung einer entwickelten Situationsmap nach Clarke [8]

Zur Auswertung des Datenmaterials wurde eine Situationsanalyse nach Clarke et al. [7] durchgeführt. Dafür wurden die Transkripte der Interviews sowie die Protokolle der teilnehmenden Beobachtung mit der Analyse-Software MAXQDA codiert. Im Zuge des offenen Codierens des Materials entstanden rund 450 Codes, anschließend wurde das Interviewmaterial in 20 Analysesitzungen durch 4 Forscher*innen bearbeitet. Relevante Elemente unterschiedlicher Lernsituationen im Umgang mit KI wurden identifiziert und Zusammenhänge auf Situationsmaps visualisiert (Abb. 1). So wurden 2 zentrale Lernpraktiken identifiziert, die im Folgenden dargestellt werden. Die Interviewzitate wurden in die deutsche Standardsprache übersetzt und geglättet.

Im Vorfeld der Datenerhebung fand ein Ethikscreening durch die Technische Universität Wien statt. Vor Interviewbeginn wurden beteiligte Personen über das Ziel der Studie aufgeklärt und anschließend um ihr schriftliches Einverständnis gebeten. Personen mit Demenz und Personen, die kein Einverständnis zur Studienteilnahme geben konnten, wurden nicht interviewt.

## Ergebnisse

Im Datenmaterial wird deutlich, dass Interesse an KI-Technologien unter Pflegeheimbewohner*innen besteht. Trotz dieses initialen Interesses werden Schulungen zwar in beiden beobachteten Fällen für Pfleger*innen und Seniorenbetreuer*innen, allerdings nicht für Bewohner*innen angeboten. Die festgestellten Lernpraktiken finden also informell und selbstgesteuert im Alltag der interviewten Pflegeheimbewohner*innen statt. Diese Lernpraktiken werden im Folgenden beschrieben (F1) und darauf aufbauend danach gefragt, welche Selbstbeschreibungen (F2) sich in Relation zu den identifizierten Lernpraktiken ausmachen lassen.

### Lernen im Umgang mit künstlicher Intelligenz

Die interviewten Pflegeheimbewohner*innen zeigen aktives Interesse daran, KI-Technologien zu verstehen: So wundert sich etwa eine Bewohnerin über den Sturzsensor, der in ihrem Zimmer angebracht wurde: „Am Anfang schaut man dauernd hin, weil es neu ist und man sich denkt: ‚Geht es einmal los?‘, und es geht nie los“ (Bewohnerin 6). Sichtbar wird durch dieses und ähnliche Beispiele eine gewisse Reflexionskompetenz [20] älterer Pflegeheimbewohner*innen im Umgang mit KI, die die Praxis der KI-Implementierung in der Pflege reflektiert und lernend damit umgeht. Weiters zeigen sich im Material neben einer solchen Reflexions- auch Ansätze einer Gestaltungskompetenz im Umgang mit KI. So beschreiben Bewohner*innen in den Interviews, wie sie KI-Technologien aktiv ausprobieren, um deren Funktionsweisen zu verstehen und zu testen, wie das Gerät in unterschiedlichen Situationen reagiert. Eine Bewohnerin schildert, wie sie sich in der Nacht über das neue Gerät in ihrem Zimmer zu wundern beginnt: „Ich liege im Bett … und schaue hinauf, und da sehe ich, dass er blinkt, und denke, ‚was ist er denn so nervös, ich tue ja gar nichts‘“ (Bewohnerin 2). Eine andere Bewohnerin beschreibt, dass sie keine direkte Rückmeldung vom Gerät erhält, aber mit dem Gedanken spielt, die Funktionsweisen des Gerätes spielerisch auszuprobieren: „Es hat sich nichts getan. Vielleicht sollte ich ja mal einen Ball werfen [lacht]“ (Bewohnerin 6). Diese Beispiele zeigen, dass ältere Pflegeheimbewohner*innen reflexiv und teilweise gestalterisch mit KI in ihrem Alltag umgehen.

### Alltags- und biografieorientierte Lernpraktiken

In der Gestaltung dieses lernenden Umgangs mit KI wird im Material weiter die Bedeutung von biografischem Wissen für das Lernen im Alter deutlich (*biografieorientierte Lernpraktiken*). So beziehen sich Bewohner*innen auf biografische Erfahrungen, um ihren Umgang mit KI zu gestalten: Eine Bewohnerin erzählt etwa, während sie mit der Robbe interagiert, von ihren Kindern oder Haustieren, um zu erklären, wie sie den Umgang mit dem Roboter handhabt: „Ich habe dran gedacht, wie das war, als wir ein kleines Schweinchen hatten. Das war auch so süß und hat sich streicheln lassen“ (Bewohnerin 5) Eine andere Bewohnerin argumentiert ihre Begeisterung für die Roboterrobbe mit ihrem lebenslangen Kontakt zu Haustieren: „Ich habe die Tierchen alle so gern“ (Bewohnerin 3). Auch im Kontext des Sturzsensors zeigt sich die Relevanz von biografischen Erfahrungen für den lernenden Umgang mit KI. So werden etwa Erfahrungen eines Sturzes zum Ausgangspunkt dafür, sich überhaupt erst mit dem Sturzsensor auseinanderzusetzen: „Ich bin leider sehr oft gestürzt. Dann haben sie mir diesen Apparat eingebaut …. Das finde ich schon sehr gut, weil es kann ja sein, dass ich in der Nacht hinfalle“ (Bewohnerin 6).

Weiter zeigt sich, dass Wissen über KI-Technologien im Alltag aufgebaut wird, etwa, indem Pfleger*innen oder andere Bewohner*innen in ihrem Umgang mit KI-Technologien beobachtet werden (*alltagsorientierte Lernpraktiken*): Bewohner 10 hat etwa gelernt, dass „mit so einer Art Schlüssel“ Alarme zurückgesetzt werden können. Eine andere Bewohnerin macht sich anhand von Fehlalarmen des Systems Gedanken darüber, wie die KI funktioniert: „Manchmal sitze ich beim Fernsehen, und die [Pfleger*innen] stürzen hinein: ‚Ist was passiert?‘ ‚Nein ich sitze da und schaue fern.‘ …Er zeigt auch an, wenn nichts ist“ (Bewohnerin 2). Diese Beispiele machen auch die Bedeutung der Relationalität im lernenden Umgang mit soziotechnischen Systemen, die als geragogisches Konzept etwa von Schramek und Engler [21] ausformuliert wurde, deutlich. Bewohner*innen ziehen bestehende Erfahrungen aus Alltag und Lebenslauf heran und lernen so „in Beziehung“ zum Selbst, zu Anderen, der Welt und technischen Geräten [13].

### Subjektivierungspraktiken

Obwohl im Material also Lernpraktiken, die im Umgang mit KI relevant werden, identifiziert werden konnten, zeigt sich auch, dass sich ältere Pflegeheimbewohner*innen nur selten als kompetente Akteur*innen im Prozess der KI-Implementierung verstehen. Vielmehr sehen die interviewten Bewohner*innen das Pflegepersonal und Technikentwickler*innen als Expert*innen im Prozess der Technikimplementierung. So verweisen Bewohner*innen in den Interviews immer wieder auf das Pflegepersonal, wenn Fragen zu KI gestellt werden: „Da weiß ich gar nichts. Haben Sie eine Schwester gefragt?“ (Bewohnerin 7).

Diese Subjektposition der Bewohner*innen als weniger kompetente Akteur*innen wird auch durch Design und Funktionsweisen des Sensors verstärkt, indem dieser so gestaltet ist, dass er für Bewohner*innen schwer erkennbar ist – und auch bei der Roboterrobbe ist der Ein- und Ausschaltknopf versteckt, sodass dieser nicht von Bewohner*innen, sondern nur von Pfleger*innen betätigt werden kann. Zum Sturzsensor beschreibt eine Bewohnerin, die Sehprobleme hat: „Da ist nur so ein schwarzer Streifen. Alles andere sehe ich nicht“ (Bewohnerin 7). Ähnliche Prozesse werden in den Interviews zur Roboterrobbe sichtbar, denn die Entscheidungsmacht zum Einsatz der Robbe liegt beim Pflegepersonal, wie eine Bewohnerin erklärt: „Die Betreuerin ist zu mir gekommen und hat gesagt, sie kommt mit der Robbe, da habe ich zugestimmt“ (Bewohnerin 9). Auch im Umgang mit dem Sturzsensor werden die Bewohner*innen kaum bis gar nicht geschult. So reagiert eine Bewohnerin auf die Frage, wozu das Gerät in ihrem Zimmer ist: „Da muss ich ehrlich sein, da kann ich Ihnen gar keine Antwort geben“ (Bewohnerin 9). Diese Selbstwahrnehmung als wenig kompetent im Kontext der Technikimplementierung stellt schließlich auch eine Herausforderung für die geragogische Unterstützung der beschriebenen Lernpraktiken dar. Wechselseitiger Austausch und eine kritische Auseinandersetzung als zentrale Bestandteile des Lernens im Alter [21] sind durch die mangelnde Einbindung von Pflegeheimbewohner*innen in die Implementierung nur schwer vorstellbar.

## Diskussion

Die Ergebnisse haben Lernpraktiken älterer Pflegeheimbewohner*innen im Umgang mit KI-Technologien aufgezeigt und problematisiert, dass diese kaum als Adressat*innengruppe von Schulungen im Kontext der KI-Implementierung verstanden werden. In der folgenden Diskussion stellen wir die Anschlussfähigkeit der Ergebnisse an Konzepte der Geragogik dar und geben einen Ausblick für weitere Forschung zum lernenden Umgang älterer Menschen mit KI:

Zunächst zeigen die Ergebnisse zu den unterschiedlichen Lernpraktiken von Pflegeheimbewohner*innen, dass Lernen im Umgang mit KI nicht isoliert, sondern relational – in Beziehung zu Pfleger*innen, technologischen Geräten und biografischen Erfahrungen – stattfindet. So greifen Bewohner*innen auf frühere Erlebnisse zurück, um die Funktionsweise der Roboterrobbe zu interpretieren, beobachten Pflegepersonal oder reflektieren ihre eigenen Sturzerfahrungen, um den Nutzen des Sturzsensors einzuordnen. Diese relationale Sicht auf Bildung und Lernen im Alter wurde in der Geragogik bereits herausgearbeitet [21] und erscheint auch anwendbar, um den lernenden Umgang von Pflegeheimbewohner*innen mit KI zu beschreiben. Gleichzeitig ermöglichen die Ergebnisse eine erweiternde Reflexion zu der Frage, wer oder was als Akteur*in im Prozess des relationalen Lernens verstanden werden kann. Hier erweitern unsere Ergebnisse bestehende Studien, die darauf hingewiesen haben, dass nicht nur in Relation zu Menschen, sondern auch im Umgang mit Materialitäten (etwa KI-Systemen) gelernt wird [19]. Inwiefern solche materiellen Akteure des Lernens im Alter zukünftig auch als lernend verstanden werden können – etwa, indem kontinuierlich Daten gesammelt und maschinell auf Basis dieser Daten gelernt wird –, stellt eine spannende Frage für weitere Entwicklung an der Schnittschnelle von Geragogik und Technik-Gerontologie dar.

Weiter verweisen die Ergebnisse auf Subjektivierungspraktiken, welche ältere Pflegeheimbewohner*innen als weniger kompetente Akteur*innen im Prozess der Technikimplementierung positionieren. Während die Bewohner*innen durchaus Wissen über die Technologien erwerben, wird ihnen wenig Raum für eine aktive Auseinandersetzung oder Mitsprache eingeräumt – etwa, indem keine Lernangebote für sie bestehen und ihre aktive Auseinandersetzung mit KI von anderen Akteur*innen übersehen wird. Diesen Befund unterstützen auch Ergebnisse zum multiperspektivischen Teil des vorgestellten Projektes, in dem neben Interviews mit Pflegeheimbewohner*innen auch Perspektiven von Technikentwickler*innen, Pfleger*innen und Angehörigen berücksichtigt wurden [6]. Dies verdeutlicht die Relevanz des geragogischen Zugangs, der neben dem Weltverständnis auch das Selbstverständnis als wichtiges Element des Lernprozesses betont [6]. Ziel von geragogischer Begleitung des KI-Umgangs im Alter sollte nicht nur ein besseres Verständnis dieser komplexen Technologien, sondern die Entwicklung von (Selbst‑)Reflexions- und Gestaltungskompetenz im Umgang mit KI sein.

Eine gezielte Förderung von Technikkompetenzen im Rahmen von KI in der Pflege stellt daher einen wichtigen Ansatz dar, um das Verständnis von älteren Pflegeheimbewohner*innen für KI-Technologien zu verbessern und es ihnen zu ermöglichen, ihre Beziehung zu KI-Technologien als aktiv Handelnde zu gestalten [21]. Partizipative Konzepte von soziotechnischer Innovation, die handelnde und lernende Menschen als Ausgangspunkt für technische Innovation verstehen, stellen hier einen förderlichen Ansatz, welcher bislang im Kontext von KI und Alter kaum angewandt wurde, dar [14]. Die Entwicklung eines dezidiert geragogischen Konzepts von KI-Innovation, gemeinsam mit entsprechenden Angeboten der Schulung und Unterstützung für ältere Menschen, könnte ein fruchtbares Feld für zukünftige Forschung sein.

Neben der praktischen Unterstützung des Lernens im Kontext von KI im Alter hat der Beitrag auch gezeigt, dass in der Forschung zu KI in der Pflege eine konzeptionelle Weiterentwicklung notwendig ist. Zu häufig werden Technologien in der gerontologischen Forschung als rein technische Interventionen verstanden, die an passive, pflegebedürftige ältere Menschen herangetragen werden [15]. Solche Stereotype, die sich im Kontext von KI teilweise noch verschärfen [23], infrage zu stellen, sollte weiteres Ziel geragogischen Arbeitens zum Thema KI sein. Auf Basis unserer Ergebnisse plädieren wir dafür, den aktiven, selbstbestimmten und mitunter widerständigen Umgang älterer Menschen mit KI als Ausgangspunkt für Lernen im Alter zu begreifen.

## Fazit für die Praxis

- Die Umsetzung komplexer technischer Innovationen in gerontologisch-geriatrischen Handlungsfeldern erfordert geragogische Begleitung, um Lernprozesse zu unterstützen.
- Die Berücksichtigung geragogischer Expertise im Prozess der Technikimplementierung kann zum Verständnis der im Alltag stattfindenden Lernpraktiken älterer Menschen beitragen.
- Die Geragogik bietet praxisrelevante Ansätze durch dialogisches Lernen und Ansätze, die ältere Menschen als aktiv Lernende im Kontext der Technikimplementierung verstehen.
